# ECCB 2012: The 11^th^ European Conference on Computational Biology

**DOI:** 10.1093/bioinformatics/bts490

**Published:** 2012-09-03

**Authors:** Torsten Schwede, Dagmar Iber

This special issue comprises the proceedings papers accepted for presentation at the 11th European Conference on Computational Biology (ECCB 2012), which was held from 9 to 12 September 2012 at the Conference Center Basel, Switzerland. This year's conference was organized by the SIB Swiss Institute of Bioinformatics and also served as the 10th [BC]^2^ Basel Computational Biology Conference, the annual meeting of the Swiss computational biology community. It was preceded by two satellite meetings, six workshops and five tutorials. Details of the conference are available through the conference website (http://www.eccb12.org) and will later be archived at eccb.iscb.org/2012/.

ECCB is the top European conference in computational biology and bioinformatics, and together with ISMB and RECOMB one of the major international conference series in computational biology. The conference brings together scientists working in a broad range of disciplines, including bioinformatics, computational and molecular biology, medicine and systems biology. It covers computational methods for analysis of the tremendously growing amount of data in the life sciences, and approaches for modeling and simulation of increasingly complex systems in molecular biology, medicine and pharmaceutical research. The scope of the conference evolves each year to address the latest developments in biological applications of mathematical modeling and computational methods.

ECCB is held in a different country or region each year, and incorporates the annual national or regional meeting where it is held. Every other year, it is held jointly with the Intelligent Systems in Molecular Biology (ISMB). Past editions of ECCB have been held in Vienna (AT) (with ISMB), Ghent (BE), Stockholm (SE) (joint with ISMB), Cagliari (IT), Vienna (AT) (with ISMB), Eilat (IL), Madrid (ES), Glasgow (UK) (with ISMB), Paris (FR) and Saarbrücken (DE).

The ECCB 2012 conference featured keynote lectures by distinguished speakers: The opening keynote lecture was delivered by Nobel price laureate Aaron Ciechanover (Technion - Israel Institute of Technology), followed by Barry Honig (Columbia University, New York), Laurent Keller (University of Lausanne), Gene Myers (MPI of Molecular Cell Biology and Genetics, Dresden), Mihaela Zavolan (Biozentrum University of Basel & SIB) and the EMBO keynote lecture by Søren Brunak (Technical University of Denmark).

The conference topics span all areas of methodological developments for computational biology and innovative applications of computational methods to molecular biology. This year, the conference was organized in 11 topic areas: ‘Applied and Translational Bioinformatics’, ‘Bioimaging, Spatial-temporal Modeling and Data Visualization’, ‘Bioinformatics of Health and Disease, Biomarkers and Personalized Medicine’, ‘Databases, Ontologies, and Text Mining’, ‘Evolution, Phylogeny, and Comparative Genomics’, ‘Macromolecular Structure, Dynamics and Function’, ‘Mutations, Variations, and Population Genomics’, ‘Protein Interactions, Molecular Networks, and Proteomics’, ‘Regulation, Pathways, and Systems Biology’, ‘Sequencing and Sequence Analysis’ and ‘Technology and Software (TechTrack)’.

Following the call for proceedings papers, we received a record number of 340 submissions—which corresponds to a ~60% increase compared to the previous ECCB in 2010. The manuscripts were assessed by a program committee of 350 reviewers and 191 co-reviewers. The reviewing process in each topic area was coordinated by two area chairs (see next article), and the different areas were coordinated by the Conference Chairs. Within each area, the area chairs assigned the papers to expert referees; in general each paper was reviewed by three PC members, with a few cases where we could only secure two referee reports. The whole process was carried out through the EasyChair conference reviewing system (http://www.easychair.org). The same system was used for discussing papers in cases where the reviewers had different opinions about the relevance or quality of the contribution. After a consensus had been reached by the reviewers, a final selection was carried out by the area chairs and the conference chairs, discussing the outcome in the form of a phone conference. In total, 48 papers were conditionally accepted, pending revision. After acceptance notices were sent, the authors had 2 weeks to modify their papers according to the suggestions made by the reviewers and to respond to the reviewers’ comments. Modified papers and authors’ responses were reexamined during the next week to ensure each paper was modified appropriately in response to the reviewers’ comments. We were pleased that all conditionally accepted papers were finally accepted. These 48 (14% accepted rate) contributions are included in this special issue. Collectively, the authors of the 48 accepted papers come from 19 countries across 4 continents, with 60 authors from North America, 95 from Europe, 27 from Asia (excluding Israel), 4 from Israel and 3 authors from Saudi Arabia. The conference papers are available in open access in electronic format, both through online open access to the journal *Bioinformatics* and on the electronic documentation distributed to the conference participants on USB memory cards.

The call for poster abstract submission was kept open 2 weeks after the selection of the oral presentations, so that authors whose manuscript could not be accepted had the opportunity to resubmit their work as poster. A call for late poster abstracts was launched to allow groups to present their latest research results during the meeting. At the time of writing, more than 500 posters were accepted after evaluation of the abstracts for scientific relevance to the meeting. The call for late abstract has not been completed at the time of writing. Accepted poster abstracts are included as part of the electronic documentation distributed to the conference participants, and are available through the conference website.

In addition to the keynotes and proceedings presentations, eight highlight presentations were selected by the ECCB steering board and program committee members from 100 submissions, consisting of full papers that have been published, or accepted for publication, between 1 January 2011 and the submission deadline, 1 July 2012. Additionally, a technology and software track included presentations of commercial and academic software, databases and biological applications by sponsors, exhibitors and academic institutions. Sixteen exhibitor stands were open throughout the conference in the main conference halls, presenting the latest scientific literature in the field of computational biology, bioinformatics, modeling and simulation, as well as new hardware, software and technology developments. ECCB 2012 also hosted the first technical meeting of ELIXIR chaired by Søren Brunak (chair of the ELIXIR Interim Board).

During the weekend preceding the conference, two satellite meetings, six workshops and five tutorials were organized: on Saturday, 8 September, a special symposium celebrated the 10th anniversary of the UniProt project: in 2002, 40 years after the first comprehensive collection of protein sequences, the ‘Atlas of Protein Sequence and Structure’, was published, the SWISS-PROT and TrEMBL protein sequence databases at the European Bioinformatics Institute (EBI) and the SIB Swiss Institute of Bioinformatics, and the Protein Information Resource (PIR) in Washington, joined forces to create UniProt. At this symposium, renowned speakers gave insight into their work, highlighting how protein databases are underpinning life sciences.

The Student Council of the International Society for Computational Biology organized its second European Student Council Symposium (ESCS), chaired by Pedro Lopes (University of Aveiro, Portugal) and Florian Heer (SIB & Biozentrum University of Basel, Switzerland). The meeting gave students the opportunity to present their work to an international audience and to build a network and exchange ideas and knowledge within the computational biology community.

On Saturday and Sunday, 8 and 9 September, the Sixth International Workshop on Machine Learning in Systems Biology (MLSB) was organized as a 2-day workshop by Karsten Borgwardt (Max Planck Institutes & Eberhard Karls Universität Tübingen) and Gunnar Rätsch (Sloan-Kettering Institute, New York). MLSB provides a scientific forum for the exchange between researchers from Systems Biology and Machine Learning.

All other workshops and tutorials were organized as 1-day events on Sunday 9 September: the workshop ‘Annotation, Interpretation and Management of Mutations (AIMM)’ focused on extraction and reuse of genotype–phenotype knowledge from scientific literature and databases. It was chaired by Christopher Baker (University of New Brunswick, Saint John, Canada) and Dietrich Rebholz-Schuhmann (European Bioinformatics Institute, Cambridge, UK). The workshop ‘Computational Proteomics: From Mass Spectrometry to Protein Complexes’ organized by Markus Müller (SIB, Geneva), Alexander Schmidt (Biozentrum, University of Basel) and Jacques Colinge (CeMM, Vienna) addressed computational aspects of Mass spectrometry analysis.

The workshop ‘Bioinformatics Training for Life Scientists: Showcases and Challenges from Tutors’ Perspectives’ organized by Patricia Palagi (SIB Swiss Institute of Bioinformatics), Vicky Schneider (European Bioinformatics Institute, EBI), Allegra Via (Dept of Physics, Sapienza University of Rome), Celia van Gelder (Netherlands Bioinformatics Centre, NBIC) focused on training in bioinformatics for life scientists from the perspective of the trainer. It aimed to provide a platform for those involved in bioinformatics training to meet, confront and share experiences, as well as discuss future directions and collaboration in joint efforts. The workshop ‘Imaging Analysis and Computational Modeling for Cancer and its Therapy’ organized by Petros Koumoutsakos and Gabor Szekely (ETHZ) presented the state of the art in imaging, analysis and modeling of cancer and its therapy. Special focus was laid on recent developments in optical microscopy, small animal and clinical imaging, data analysis tools, mathematical models and computations of cancer formation and progression. The workshop ‘Detecting Transcription Factor Binding Sites with ChIP-Seq Data and Predicting Damaging Cis-Regulatory Variations’ addressed genome re-sequencing data analysis for the prediction of variations within *cis*-regulatory sequences. The program was organized by Wyeth W. Wasserman (University of British Columbia, Canada), Virginie Bernard (Curie Institute, Canada), Jonathan Lim and Anthony Mathelier (University of British Columbia, Canada).

The purpose of the tutorial program at ECCB is to provide participants with lectures and hands-on training covering topics relevant to the bioinformatics field. It offers participants an opportunity to learn about new areas of bioinformatics research, to get an introduction to important established topics, or to develop advanced skills in areas they are already familiar with. At ECCB 2012, tutorials on six different topics were organized: ‘Applications of Bio-Ontologies in Large-Scale Data-Driven Science: A Practical Introduction’ by Barry Smith (University at Buffalo, USA), and Janna Hastings (European Bioinformatics Institute and University of Geneva); ‘Protein Evolution: From Sequence to Structure to Function’ by Christine Orengo and Romain Studer (UCL, London, UK) and Nicholas Furnham (EMBL-EBI, Cambridge, UK); ‘GWAS: Statistics and Bioinformatics to Analyse Genotype to Phenotype Relationships from Molecular Traits to Disease Phenotypes’ by Christoph Lippert (Microsoft Research, Los Angeles, USA) and Oliver Stegle (Max Planck Institute for Developmental Biology, Tübingen, Germany); ‘Inferring Genetic Diversity from Next-generation Sequencing Data: Computational Methods and Biomedical Applications’ by Niko Beerenwinkel (ETH Zurich and SIB), Karin J. Metzner (University Hospital Zurich) and Volker Roth (University of Basel, Switzerland); and ‘Reads to Biological Patterns: End-to-End Differential Expression Analysis of RNA Sequencing Data Using Bioconductor’ by Simon Anders and Wolfgang Huber (EMBL), Michal Okoniewski (ETHZ) and Mark Robinson (University of Zurich & SIB).

Encouraging the participation of young scientists has always been a central goal of the ECCB conference because the conference is a great opportunity for education and networking for early stage scientists. Thanks to contributions from several academic sponsors, our host organizations, previous editions of ECCB and our commercial sponsors, we could provide 114 conference fellowships to PhD and postdoc applicants from all over the world. Presenters and contributors to oral presentations were given priority. Unfortunately, several applicants had to be turned down due to limitations of available resources.

Organizing ECCB would not be possible without the generous support by partner organizations, funding agencies and sponsors. We are especially grateful to the Swiss Foundation for Excellence and Talent in Biomedical Research, SIB Swiss Institute of Bioinformatics, ISCB International Society for Computational Biology, the University of Basel, the Swiss Academy of Medical Sciences and the [BC]^2^ Basel Computational Biology Conference for funding 114 fellowships to facilitate the participation of young talented scientists early in their career. We highly appreciate the support by our host institutions, the SIB Swiss Institute of Bioinformatics, the Biozentrum University of Basel, and the Swiss Systems Biology Initiative SystemsX.ch. We are especially grateful to our platinum sponsors Basel Area and IBM. We would like to thank all our partner and sponsors: the Netherlands Bioinformatics Centre NBIC, CONVEY Computers, F. Hoffmann-La Roche Ltd, HP, BBRC, Interpharma, FMI Friedrich Miescher Institute, Bioalps, Competence Center Computational Sciences University Basel, Novartis Pharma, Syngenta, BASF, Merck Serono and the canton of the city of Basel. We would like to thank EMBO for supporting the EMBO keynote lecture by Soren Brunak.

It is our pleasure to thank all the people and organizations that made ECCB 2012 possible. Our first thanks go to the program committee of reviewers and co-reviewers, and in particular the area chairs. A swift and effective review process is at the heart of a conference like ECCB. Reviewing 340 proceedings manuscripts and 100 highlight paper submissions in just a few weeks is truly challenging, especially since many members of the program committee had been involved in reviewing for ISMB shortly before. The dedication and hard work of the program committee was quite simply the key to the high quality of the conference.

We are grateful to the ECCB steering committee for their support to the organization of the conference: in particular, the advice and guidance from previous organizers Anna Tramontano, Yves Moreau and Michal Linial was crucial. Anna, thank you so much for answering our questions at any time of day (and night)!

Logistics is crucial for a successful conference. We are grateful to the Oxford University Press production team for the preparation of this special issue, to Congress Center Basel for the venue organization. The local administration team at the Biozentrum of the University of Basel did an incredible job, and we owe them a great debt: Katja Jenni, Rita Manohar, Sarah Güthe, Lorenza Bordoli and Yvonne Steger for preparing the meeting and organizing the registration, Markus Stöckli from Billboard for the poster and website design, Andrew Waterhouse for the registration and poster submission system, JanWelker and Konstantin Arnold for IT support, Jocelyne Boquet for handling the financial administration.

Finally, the most important ingredient to the success of a conference are the scientific contributions and discussions: we are grateful to the authors of all submitted manuscripts, keynote speakers, presenters of selected papers, tutorial lecturers, workshop organizers and contributors, poster authors and to all the participants. Thank you for making this conference a success.

